# Monocular 3D Human Pose Markerless Systems for Gait Assessment

**DOI:** 10.3390/bioengineering10060653

**Published:** 2023-05-26

**Authors:** Xuqi Zhu, Issam Boukhennoufa, Bernard Liew, Cong Gao, Wangyang Yu, Klaus D. McDonald-Maier, Xiaojun Zhai

**Affiliations:** 1 School of Computer Science and Electronic Engineering, University of Essex, Colchester CO4 3SQ, UK; xz18173@essex.ac.uk (X.Z.); ib20472@essex.ac.uk (I.B.); cg21670@essex.ac.uk (C.G.); kdm@essex.ac.uk (K.D.M.-M.); 2 School of Sport, Rehabilitation, and Exercise Sciences, University of Essex, Colchester CO4 3WA, UK; bl19622@essex.ac.uk; 3 The Key Laboratory of Intelligent Computing and Service Technology for Folk Song, Ministry of Culture and Tourism, Xi’an 710119, China; 4 School of Computer Science, Shaanxi Normal University, Xi’an 710119, China

**Keywords:** computer vision, deep learning, markerless, gait analysis, Kalman filter, monocular camera

## Abstract

Gait analysis plays an important role in the fields of healthcare and sports sciences. Conventional gait analysis relies on costly equipment such as optical motion capture cameras and wearable sensors, some of which require trained assessors for data collection and processing. With the recent developments in computer vision and deep neural networks, using monocular RGB cameras for 3D human pose estimation has shown tremendous promise as a cost-effective and efficient solution for clinical gait analysis. In this paper, a markerless human pose technique is developed using motion captured by a consumer monocular camera (800 × 600 pixels and 30 FPS) for clinical gait analysis. The experimental results have shown that the proposed post-processing algorithm significantly improved the original human pose detection model (BlazePose)’s prediction performance compared to the gold-standard gait signals by 10.7% using the MoVi dataset. In addition, the predicted *T*^2^ score has an excellent correlation with ground truth (*r* = 0.99 and *y* = 0.94*x* + 0.01 regression line), which supports that our approach can be a potential alternative to the conventional marker-based solution to assist the clinical gait assessment.

## 1. Introduction

Gait impairments are common in many medical conditions [1,2], which have the potential to modify clinical symptoms, alter the energy cost of movement, and negatively affect the quality of life [3,4]. Clinical gait analysis plays an important role in the quantification of these impairments, as such information may be used for clinical decision making [5,6] and the design of new therapies.

Traditionally, the most common, accurate and reliable measurement systems used for clinical gait analysis are optoelectronic motion capture systems [7]. However, these systems cannot be easily used in real-world environments because they are expensive, not easily portable, and rely on trained personnel for assessment. In recent years, inertial measurement units (IMUs) became an alternative solution for clinical gait analysis [8]. However, IMUs may not be an ideal alternative to optoelectronic systems, as they require time for sensor placement and they are sensitive to environmental conditions, and the sensors in the IMU may gradually deviate from their initial calibrated values [9].

For clinical gait analysis to be translated ubiquitously in the clinics and fields, there is a need for methods that are cost effective, require limited time for equipment set-up and processing and do not rely on specialist personnel for assessment.

Markerless motion capture uses standard video to record movement without markers, often leveraging deep learning-based software to identify body segment positions and orientations (pose). Currently, there are free two-dimensional (2D) motion analysis software tools, such as Kinovea [10], which can estimate 2D human kinematics using videos captured by a single camera. However, these systems rely partially on human annotation of anatomical landmarks. Other solutions adopt multi-cameras [11] or depth cameras [12] to analyse kinematics on reconstructed 3D human postures, but restrict subjects to collecting gait data in specific experimental settings and a large laboratory space. Recent progress in the field of computer vision and deep learning provide powerful human pose detection models to reconstruct 3D human posture by estimating the joint locations in 2D videos [13], making it possible to create a holistic markerless gait analysis system with a monocular camera. In addition, Liang’s study [14] indicates that this technique allows gait analysis to be performed without specific experimental demands, which is particularly useful for mobility-impaired patients. Therefore, this paper will introduce a cost-effective and markerless gait assessment system using a human pose detection model.

**Figure 1:**
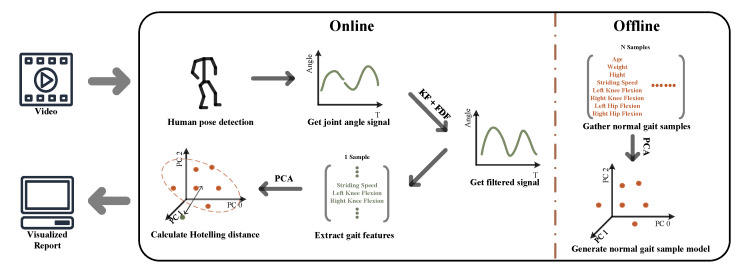
Monocular camera marker-less gait analysis system; PCA (Principal Components Analysis), KF (Kalman Filter), FDF (Frequency Domain Filter).

In our previous work [15], we developed a markerless, non-invasive rehabilitation assessment system using a consumer monocular camera together with a human pose detection model, BlazePose [16], to assess the patient’s gait in an indoor environment that only requires a limited number of gait cycles (i.e., 2–3 strides for a video sample). Although there are other models such as OpenPose [17], D3KE [18] and 3DPoseNet [14] providing better accuracy in human pose estimation tasks, Refs. [18,19] suggest that the BlazePose model has a faster runtime performance and lightweight nature, which allows for the integration of additional smoothing algorithms and gait-oriented evaluation algorithms on mobile devices. The processed results can then be used to assist healthcare workers with the creation of a personalised rehabilitation plan for patients with gait impairments. As illustrated in Figure 1, the system processes walking video by detecting the human pose, receiving gait signals, filtering gait signals, extracting discrete gait features and computing the Hotelling distance (i.e., $T^{2}$ score) in succession. In this paper, we improve the filter strategy to obtain a better-quality gait signal. In addition, the system performance was validated by comparing filtered gait signals, discrete gait parameters, $T^{2}$ score prediction, and normal gait sample model principal components with ground truth provided by a marker-based motion capture camera system.

This paper aims to further validate the optimised system’s performance in terms of the predicted gait signal, predicted discrete gait parameters, predicted $T^{2}$ score, and predicted normal gait sample models by employing a public dataset. Regarding the experiments’ results, the markerless solution does not provide the same level of performance metrics compared to the traditional marker-based method; however, the predicted $T^{2}$ score has an excellent correlation with the ground truth. Therefore, this new approach can be used as a cost-effective alternative solution to the conventional marker-based solution, aiding professionals by providing an initial clinical report. In addition, the markerless solution is built on BlazePose and a signal 2D camera instead of using multi-camera and motion capture markers, which makes it possible to deploy this assessment system on mobile devices in the future.

The main contributions of this paper can be summarised as follows: (1) The filter strategy in the post-processing stage has been optimised to improve the joint angle signal prediction accuracy by 10.7% when compared with the raw joint angle signal predicted from BlazePose. (2) A public dataset is used to validate the performance of the system from various perspectives, which can be used to provide a comparative benchmark for other similar work. (3) We further investigate the possibility of developing a low-cost clinical gait analysis system based on BlazePose by evaluating and analysing its pros and cons.

This paper is organised as follows: the system details are introduced in Section 2. Section 3 presents the corresponding evaluation metrics, dataset, and experiment results for system validation. In Section 4, the conclusions are drawn.

## 2. Methodology

The assessment system first converts video input into the gait signal and decodes the gait parameters to obtain the visualised report. In this section, the technical details are briefly introduced to cover the assessment system’s three primary functions:

1. Generating gait signals by computing joint angles.
2. Processing gait signals to complement the missing signals and removing any noise from the signals.
3. Creating a feature model by extracting the discrete gait parameters from gait signals.

### 2.1. Generate Gait Signals

In this study, the angles of the knee and hip are treated as the main gait signals. They may be interpreted by the joint angle and the discrete time domain signal, respectively. We focus on the analysis of sagittal plane joint angles because it provides more insightful information in the clinical gait analysis context [11]. As a result, a camera view perpendicular to the walking direction is preferred. The discrete-time domain signal suggests that the gait signal is stacked by the joint angles from each frame in chronological order.

For the first function, the assessment system requires the user to input video data and predicts human poses to generate the gait signals. The BlazePose human pose detecting model returns the three-dimension joint coordinates, as well as the corresponding visibility level (i.e., a percentage score for the confidence of joints’ prediction in each image). Those joint coordinates can be converted into vectors, and the desired joint angle can be obtained by applying cosine law to the vectors.

**Figure 2:**
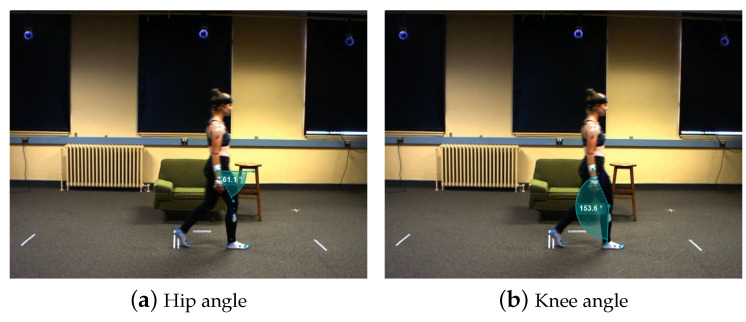
Joint angle definition diagram [10,20].

Figure 2 illustrates the definitions of the hip and knee angles in this study, where the angle between knee-to-hip and knee-to-ankle vectors is defined as the knee angle.

However, the hip angle is defined as slightly different from the knee angle. Based on the Cosine law, $\theta =arccos\frac{\vec{a}\cdot \vec{b}}{\left|\vec{a}\right|\left|\vec{b}\right|}$, it yields a smaller angle ($\theta \leq 180^{\circ }$). While simply using hip-to-shoulder and hip-to-knee vectors to calculate the hip angle, it will gradually increase to $180^{\circ }$ and abruptly decrease from $180^{\circ }$ when the shoulder, hip, and knee are almost in the same line, which affects the subsequent filtering process. So, instead of using hip-to-shoulder and hip-to-knee vectors, the virtual vector lying parallel to the moving direction is applied to construct the hip angle. In principle, the virtual vector can be calculated by rotating the left shoulder to the right shoulder vector by $90^{\circ }$ via the vertical axis.

### 2.2. Post-Process Gait Signals

Gait signals generated by a simple angle calculation suffer from data loss and noise signals due to many elements, including low video resolution, the overlapping nature of low limbs, errors in the human pose detection model, as well as other unknown factors in the practical environment; for example, insensitivity of the human posture model to certain clothing or postures caused by the training sample selection preferences. We define such gait signals as raw or original joint angle signals. To further improve the signal quality, a post-process with the KF and FDF was employed.

The KF algorithm provides an efficient recursive method to estimate the state of a process [21]. In addition, Sam and Jill’s work [21] suggests that KF can be adopted to smooth gait signal. To apply KF in gait signal filtering, the state-transition matrix *A*, the state estimation matrix *S*, the measurement matrix *H*, and the state transition equation are represented as follows.

(1) $$\begin{array}{cc} A & =\left[\begin{array}{ccc} 1 & \Delta t & \frac{\Delta t^{2}}{2}\\ 0 & 1 & \Delta t\\ 0 & 0 & 1 \end{array}\right]\;S=\left[\begin{array}{c} \theta \\ v\\ a \end{array}\right]\;H=\left[\begin{array}{ccc} 1 & 0 & 0 \end{array}\right] \end{array}$$

(2) $$\begin{array}{cc} \; & {\hat{S}}_{t|t-1}=A{\hat{S}}_{t-1|t-1}+{Pnoise}_{t} \end{array}$$

(3) $$\begin{array}{cc} \; & {\hat{\theta }}_{t|t}=H{\hat{S}}_{t|t}+{Mnoise}_{t} \end{array}$$

where the state estimation matrix *S* in Equation (1) contains joint angle θ, angular velocity *v* and angular acceleration *a*. The Pnoise and Mnoise refer to processing noise and measurement noise obeying Gaussian distribution Pnoise∼(0,Q) and Mnoise∼(0,R), respectively. Specifically, *Q* is the process noise covariance matrix and *R* is the measurement noise covariance matrix. The posterior joint angle estimate ${\hat{\theta }}_{t|t}$ is obtained using Equations (2) and (3).

The FDF uses Discrete Fourier Transform (DFT) and Inverse Discrete Fourier Transform, supported by a suitable filter strategy, to select principal frequency components to recover the filtered signal. KF and FDF were both used in earlier work [15] to predict missing data and denoise the original gait signals. Studies have demonstrated that KF and FDF can help reduce assessment system failures caused by the above problems. In this paper, we further optimise the strategy and provide further details on this in the upcoming sections.

The first optimisation we used was to address the KF failure when joint angles are missing on non-linear change periods. The KF-based approach uses the classical linear kinematic equations to model the variation in joint angle, which is established on the assumption that the joint angle signal varies approximately linearly over a short time. Most of the time, this algorithm is reliable; however, from the perspective of the entire gait signals, the joint angle signal during walking is non-linear but could be seen as the combination of multiple cosine signals. Additionally, the experiments illustrating the KF algorithm still struggle with prediction when the data are missing. This is due to the low level of visibility (≤40%).

**Figure 3:**
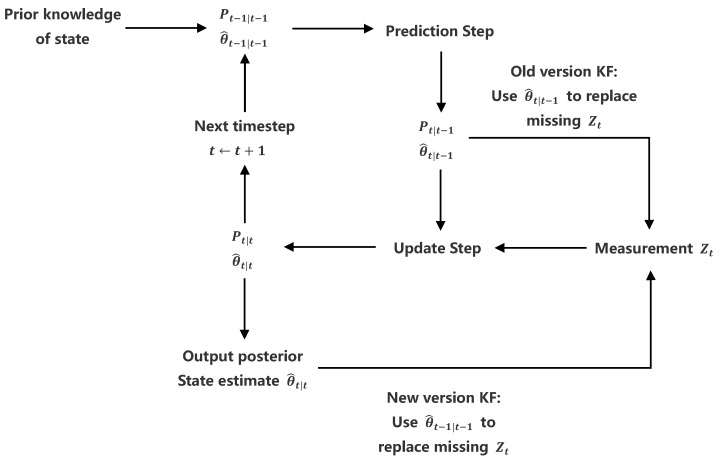
Missing measurements replacement strategy [15,22].

In this paper, we improve the missing measurements’ replacement strategy to trace the non-linear angle changes in low-visibility conditions. The details of the strategy are illustrated in Figure 3, where the ${\hat{\theta }}_{t|t-1}$ and ${\hat{\theta }}_{t|t}$ represent the prior and posterior joint angle estimate, respectively. Similarly, $P_{t|t-1}$ and $P\theta_{t|t}$ are the prior and posterior state estimate covariance, respectively. In the prior version KF, the prior state estimate ${\hat{\theta }}_{t|t-1}$ obeying the classical linear kinematic model was used to replace the current missing measurement $Z_{t}$, which resulted in the predicted joint angle gradually deviating from the true value, especially for the successive measurements lost during the non-linear change interval. A possible alternative solution is to use the posterior joint angle estimate ${\hat{\theta }}_{t-1|t-1}$ to substitute the current missing measurement $Z_{t}$, which prevents KF being excessively reliant on the predicted results.

**Figure 4:**
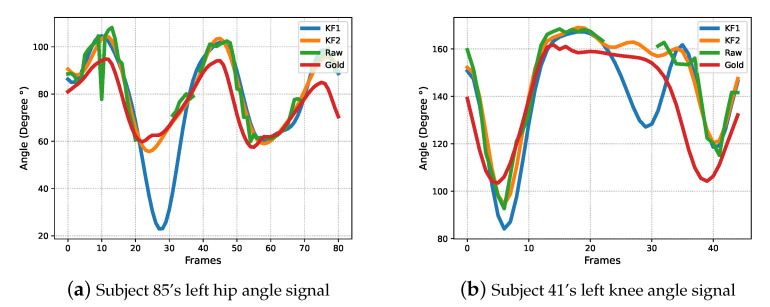
Earlier version KF ($KF_{1}$) V.S KF with optimisation ($KF_{2}$).

Figure 4 shows a comparison of the new version $KF_{2}$ with the prior version, and how it reduces the distortion of predicted gait signals in low-visibility environments. The prior version $KF_{1}$ failed to trace the non-linear joint angle signals when the visibility level dropped below the threshold (≤40%). However, the new version KF corrects the prediction by inputting the posterior joint angle estimate $\theta_{t-1}$ as the current missing measurement $Z_{t}$.

The second optimisation we designed aimed to improve the KF performance in terms of prediction accuracy. In this paper, a set of videos with the targets’ sagittal plane is applied to evaluate the assessment system. When a monocular camera is used to capture sagittal plane movements, the far-side joints suffer from severe obstruction, which causes the joint angle signal and the near-side joint angle signal to exhibit different process noise Pnoise and observation noise Mnoise. Therefore, instead of using shared process noise and observation noise in the earlier work, two sets of noise covariance matrices are found for joint signals on each side of the sagittal plane, respectively.

After the KF estimates the missing gait signals, the FDF using the Least Root Mean Square Error strategy (LRMSE) [15] will be used to calculate the principal frequency components and then recover them to the time domain gait signal. Additionally, previous research [15] suggested using the pose segmentation mask to reduce the background noise level. The KF and FDF algorithm complement and denoise original gait signals and return a processed gait signal. This step is called “post-process gait signals”.

### 2.3. Gait Parameters Analysis

Even though we are able to obtain the gait signals from markerless video inputs, the original signals with hidden features are still abstract and unreadable, making them difficult for non-specialists to analyse. In this paper, we propose a gait signal decoding function, which processes the data to produce discrete gait metrics, including joint flexion and striding speed. These discrete gait parameters are separately stored in different datasets in terms of the targets’ age and gender, which can later be used to create models for analysis, called “feature models” [15]. This function enables the assessment system to evaluate patients’ disease condition with a $T^{2}$ score for aiding healthcare workers, even non-specialists, to learn about patients’ recovery progress and make suitable clinical decisions.

To design this function, PCA was utilised to generate feature models to hold sample features. Suppose a dataset is a $\left(n,d_{1}\right)$ matrix that contains *n* samples, each with $d_{1}$ features, including their gait parameters and physiological indicators (e.g., age, mass, and height). The PCA algorithm will identify a series of principal components (PCs) representing *n* samples’ information through *n* vectors, with $d_{2}$ dimensional ($d_{2}\leq d_{1}$), which is known as the feature model, while explaining 90% of the samples’ variance. Once the feature models were generated, the assessment system can report the Hotelling distance ($T^{2}$ score) to indicate the difference in gait between the patient and the normal group samples; the higher the $T^{2}$ score, the more irregular the patient’s gait. The filtered gait signal, discrete gait parameters, and predicted $T^{2}$ score construct the system’s gait analysis report, validated, respectively, in the next section.

## 3. Experiment Results

A series of experiments were designed to verify the assessment system’s performance. The experiments focused on the following three points: (1) they evaluated the improvements in performance for the optimised assessment system, (2) they assessed the agreement between the conventional method and the markerless method for the discrete gait parameters, and (3) they evaluated the predicted feature model and the correlation of the $T^{2}$ scores to the gold-standard.

Four topics will be discussed sequentially in the following section. Firstly, Section 3.1 introduces metrics used in this study for evaluating the system performance. Secondly, Section 3.2 introduces the details of the dataset. Thirdly, Section 3.3 shows the settings of the hyper-parameters in the assessment system. Finally, the experiments’ findings are reported in Section 3.4, to validate the assessment performance.

### 3.1. Evaluation Metrics

To objectively assess the system’s performance, a range of metrics was introduced to validate the markerless assessment system performance from three perspectives: (1) the accuracy of filtered gait signal, (2) the agreement between predicted and gold-standard discrete gait parameters, and (3) the correlation of $T^{2}$ score between assessment system prediction and gold-standard.

(4) $$\begin{array}{cc} PE & =\frac{1}{S}\sum_{s=1}^{S}\frac{1}{J}\sum_{j=1}^{J}\frac{\sqrt{\frac{1}{F}\sum_{t=1}^{F}{\left(q_{s,j}\left(t\right)-q_{gt,s,j}\left(t\right)\right)}^{2}}}{max\left(q_{gt,s,j}\right)-min\left(q_{gt,s,j}\right)} \end{array}$$

(5) $$\begin{array}{cc} DTW(G_{i},P_{j}) & =Cost\left(G_{i},P_{j}\right)+min\left\{\begin{array}{cc} & DTW(G_{i-1},P_{j})\\ & DTW(G_{i-1},P_{j-1})\\ & DTW(G_{i},P_{j-1}) \end{array}\right. \end{array}$$

There are two accuracy performance metrics used to describe the markerless gait signals: the percentage error (PE) [23] and Dynamic Time Warping (DTW) distance [24]. F. De Groote suggests using the ratio of cumulative Root Mean Square Error for all points against the corresponding joint’s flexion, PE (Equation (4)), to evaluate the similarity of the two signals, where *S* is the number of samples. The number of joint angles is represented by the parameter *J*. The number of frames for the corresponding sample is represented by the parameter *F*. The gait signal for the sample *s* of joint *j* is represented by $q_{s,j}$. The DTW distance is commonly used to compare the similarity of speech signals with varied lengths. However, Yu and Xiong [25] suggested using the DTW distance to assess physical rehabilitation, which encouraged us to apply it to assess the system’s performance. The DTW distance, according to Equation (5), uses a dynamic programming method to find the optimal warping path, which has the lowest cumulative cost of matching each point in two signals [25]. Suppose we observe signal *G* and the predicted signal *P*, both with a length of *m*, and $DTW(G_{i},P_{j})$ denotes the distance of the best warping path between *G* and *P* from (1,1) to (i,j), where 1≤i≤m, 1≤j≤m. The $Cost(G_{i},P_{j})$ treats Euclidean distance as the cost of matching point $G_{i}$ and $P_{j}$. In this paper, the DTW distance was normalised using signal length (2m) as the final result.

In addition to assessing the accuracy of the gait signals, the Bland–Altman plot analysis [26,27] was introduced to evaluate the agreement of the discrete gait parameters between markerless and marker-based measurement methods. The linear regression-based method, Person correlation coefficient (r), and cosine similarity were employed, respectively, to assess the correlation between gold standards and predicted results, such as striding speeds, $T^{2}$ scores, and the feature model’s principal components obtained by PCA.

### 3.2. Datasets

In an earlier work [15], a small dataset containing nine samples was employed to briefly evaluate the performance of the markerless system and demonstrate the system’s basic functionality. To further validate the vision-based markerless assessment system, we pre-processed (i.e., extracted a walking interval from original videos and calculated the corresponding gold-standard) 78 samples provided by MoVi [20] to compose a robust dataset with more diverse samples. Table 1 illustrates the details of those samples.

Movi publishes a series of samples with 30 FPS and 800 × 600 pixels video and corresponding joint locations, acquired by a stationary computer vision camera binding with Qualisys Track Manager (QTM) software and Visual3D software. In the pre-processing stage, the joint locations will be converted into gold-standard gait signals. Overall, 78 samples were selected from sequence F_PG1 (total 87 samples in F_PG1), including 50 female and 28 male samples, which excludes the samples with an excessively short walking period (i.e., less than 40 frames) and the samples with obvious errors in the gold-standard gait signal. These samples were then clipped in accordance with the interval. To obtain the sagittal plane walking periods, the intervals began with the targets’ entry into the walking state (i.e., taking the first step) and ended with their last step before entering the standing state. These periods form the datasets for testing the markerless assessment system.

### 3.3. Hyper-Parameters Setting

There is a range of hyper-parameters that can directly affect the performance of the markerless assessment system, including segmentation mask (SM), min detection confidence (MDC), and min tracing confidence (MTC), which are provided by BlazePose as the initialisation parameters. The SM enables Blazepose to reduce the background noise level in the video to aid human pose detection. MDC and MTC, according to previous research [15], seemed to have less effect on human pose prediction results. Therefore, SM is set to active (SM = True) and (MDC = 30% MTC = 50%) to follow the configuration from the previous work. In addition to the above initialisation parameters, the visibility threshold (VT) is another important parameter that should be carefully selected. Earlier studies have shown that VT = 40% is a suitable option for the post-processing [15]. When BlazePose predicts the joints in the frame with a visibility level lower than VT, it means the system assumes that the targets are lost and the corresponding joint angles will be predicted by the KF algorithm.

Additionally, the FDF needs to designate cut for dropping a portion of the KF predicted gait signal at the beginning, since the KF Iteration requires time to converge. It is necessary to specify the filter strategy and how many frequency components (*N*) are used to recover the time domain gait signal. According to the previous studies [15], cut=10% of the prefix will be removed from signals, and Least Root Mean Square Error (LRMSE) strategy is adopted to select N=5 principal frequency components that can recover a time-domain signal with the lowest Root Mean Square (RMS) error for denoising the gait signals.

(6) $$\begin{array}{cc} Q & =\alpha \left[\begin{array}{ccc} 1 & \cdots & 0\\ \vdots & \ddots & \vdots \\ 0 & \cdots & 1 \end{array}\right]R=\beta \left[\begin{array}{ccc} 1 & \cdots & 0\\ \vdots & \ddots & \vdots \\ 0 & \cdots & 1 \end{array}\right] \end{array}$$

(7) $$\begin{array}{cc} P_{0} & =\left[\begin{array}{ccc} 100 & \cdots & 0\\ \vdots & \ddots & \vdots \\ 0 & \cdots & 100 \end{array}\right] \end{array}$$

In addition to preserving the configurations of the previous hyper-parameters in [15], dedicated coefficients α and β are both required to calculate the Pnoise and Mnoise matrices for the KF to predict distal and proximal joints, respectively, where the processing noise obeys the Gaussian distribution Pnoise∼(0,Q) and the measurement noise obeys the Gaussian distribution Mnoise∼(0,R). The process noise covariance matrix *Q*, the measurement noise covariance matrix *R*, and the error estimate covariance matrix *P* at t=0, $P_{0}$ are set as per Equations (6) and (7).

Table 2 illustrates the sum of the average PE and normalised DTW distance from KF-predicted knee and hip angle signals for the two observed distances within 13 different α and β combinations. Ideally, the combinations with the lowest percentage error and the shortest DTW distance are preferred; however, it is rare to have both. The results indicated that independent noise covariance matrix allows the KF to cope better with mixed-noise gait signals, and the trade-off coefficients combination ($\alpha_{far}=1\times 10^{-2}$, $\beta_{far}=1\times 10^{-3}$) and ($\alpha_{near}=1\times 10$, $\beta_{near}=1$) has a low PE and a short DTW distance.

To summarise, the hyper-parameters of the markerless assessment system are set as follows: MDC=50%, MTC=30%, VT=40%, SM=TRUE, cut=10%, $\alpha_{far}=1\times 10^{-2}$, $\beta_{far}=1\times 10^{-3}$, $\alpha_{near}=10$, $\beta_{near}=1$, N=5 and the filter strategy is LRMSE.

### 3.4. System Performance Evaluation

In this section, the performance of the system is validated from three different perspectives: (1) the predicted gait signals’ accuracy compared to the gold-standard gait signal, (2) the agreement between the motion capture markers method and the markerless method for the discrete gait parameters, and (3) the correlation between the gold-standard and assessment system prediction for striding speeds and feature model ($T^{2}$ scores).

Table 3 shows the PE and DTW distance of the original gait signals, the signals processed by the prior version KF ($KF_{1}+FDF$), and the signals processed by the KF with two optimisations mentioned in this paper ($KF_{2}+FDF$). Additionally, rather than classifying gait signals according to the physiological position of the right and left limbs, Table 3 evaluates gait signals in terms of the distance of the lower limbs (far-side/near-side) to reflect the system performance under low-visibility conditions. To avoid overstating errors in the original signals, the missing data ‘None’ are substituted by the mean value of the original signals or the most recent valid value, when computing PE or DTW, respectively.

Comparing $KF_{2}+FDF$ with the original signals, the DTW distances are significantly reduced for far knee, far hip and near hip angle signals while maintaining similar or even lower PE, indicating that the updated KF ($KF_{2}$) can effectively improve the similarity of the predicted signals to the ground truth without introducing an additional DC component offset of the signal or global offset. More specifically, compared to the original signals, the average DTW distance of $KF_{2}+FDF$ decreases 10.7% ((3.64−3.25)/3.64); however, the average PE remains at the same levels (25.57% V.S. 27.77%). In addition, Figure 4 indicates that the new version $KF_{2}$ has better robustness when occasional transient missing data occurs, compared with $KF_{1}$. Specifically, the $KF_{2}$ improves subject 85’s left hip signal and subject 41’s left knee signal by 68.7% and 37.5% at DTW distance (i.e., $\left(DTW\left(GoldStandard,KF_{2}\right)-DTW\left(GoldStandard,KF_{1}\right)\right)/DTW\left(GoldStandard,KF_{2}\right)$), respectively.

**Figure 5:**
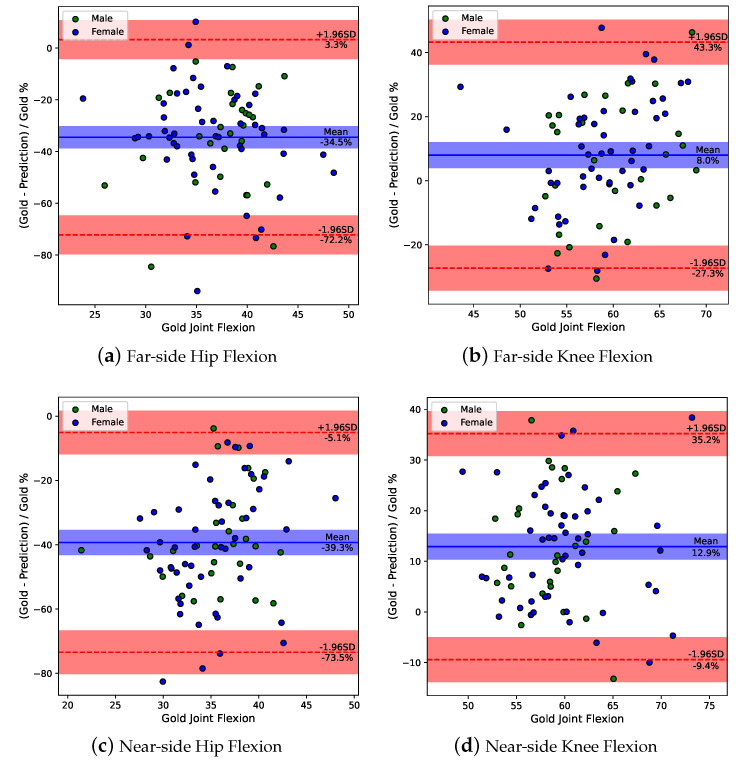
Bland–Altman plots for four joints’ flexion. (**a**) *p*-value = 0.40; (**b**) *p*-value = 0.84; (**c**) *p*-value = 0.90; (**d**) *p*-value = 0.93.

Figure 5 illustrates four joint flexion Bland–Altman plots. It is more interesting to discuss distal/proximal than left/right joint flexion angles because the targets’ discrete gait parameters obtained from the markerless assessment system are sensitive to the visibility level. The Kolmogorov–Smirnov test proves that the differences obey the normal distribution (*p*-value > 0.05). The blue horizontal line indicates the mean difference between the traditional method and the markerless method. Suppose the mean difference is ($\bar{d}$) and the standard deviation of the differences is (sd), 95% of differences will be located in the region between the red dashed line ($\bar{d}-1.96sd$ to $\bar{d}+1.96sd$), while the blue and red shading area suggests the potential values (95% confidence interval) of the real mean and real 95% boundaries of the overall sample estimated from a finite sample. The gap between the blue horizontal line and the red dashed line represents the limit of agreement (LoA).

The near-side flexion exhibits higher variation in the mean difference (knee mean = 12.9%, hip mean = 39.3%) compared with far-side flexion (knee mean = 8.0%, hip mean = 34.5%). However, not unexpectedly, the far-side flexion (hip LoA = 37.7%, knee LoA = 35.2%) suffers more uncertainty difference in individual samples than near-side flexion (hip LoA = 34.2%, knee LoA = 22.3%). This supports that the obscured far-side limb results in a larger deviation in the markerless prediction. Additionally, the predicted joint flexions have 8.0% to 39.3% mean difference compared with ground truth, and none of the blue shadow areas contain y=0, which indicates the markerless method based on BlazePose exhibits statistically significant differences from the traditional method using a motion capture marker.

The striding speed (i.e., the number of strides per second) and $T^{2}$ score reject the null hypothesis (*p*-value < 0.05). In this case, the linear-regression-based method and Person correlation coefficient (*r*) are introduced to evaluate the system performance. To fully utilise the limited data, the cross-validation strategy is employed, in which each sample is individually selected as the test subject to compute the $T^{2}$ score and the remaining samples are used to create the feature model.

**Figure 6:**
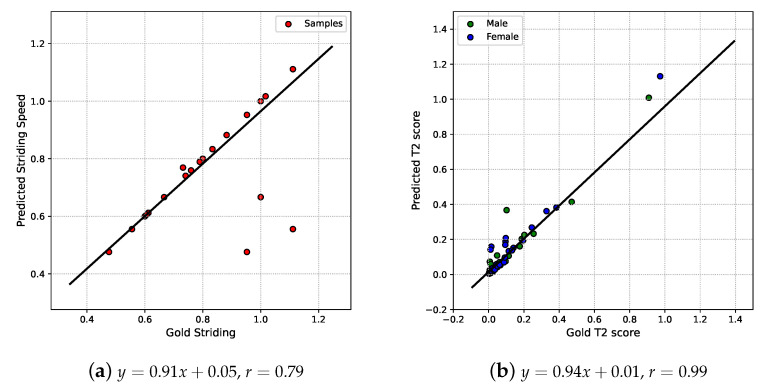
Striding speed (**a**) and $T^{2}$ score (**b**)’s correlation.

The striding speeds plot with r=0.79 and y=0.91x+0.05 regression line is illustrated in Figure 6a. Although the correlation coefficient suggests that markerless prediction does not appear to be a desirable gold-standard alternative, 92% of samples are located on the ideal regression line y=x. This phenomenon may derive from the prediction method: we treat the PC’s frequency in the near-side hip angle signal as the striding speed, which means the predicted results are sensitive to the gait signals’ shape. However, 92% accuracy is satisfactory for markerless predicted striding speed.

Figure 6b illustrates that the two methods’ $T^{2}$ scores have an r=0.99 and y=0.94x+0.01 regression line. This indicates that the usage of age, weight, height, predicted joint flexions, and predicted striding speed seems to be a good substitute for discrete gait parameters measured by the traditional method to build a feature model. However, the $T^{2}$ scores are clustered in the (0–0.4) region, which makes the differences between the two methods apparent. In order to clearly assess the similarity of the feature models obtained using the two methods, cosine similarities for the first four PCs in the gold-standard feature model and predicted feature model are used and listed in Table 4.

The $T^{2}$ scores in Figure 6b are computed by the female or male feature models, respectively, in terms of the target’s gender. Therefore, Table 4 lists two feature models’ PCs. The ‘Explained variance‘ in Table 4 denotes the weight of the corresponding PC in their feature model, and the cosine similarity denotes the similarity of $PC_{i}$ between the predicted and gold-standard feature model. As shown in Table 4, the predicted female feature model has relatively good similarity to the ground truth, while the predicted male feature model has an unacceptable similarity. A possible reason is that the male sample is insufficient in size (28 male samples V.S 50 female samples). Although the Bland–Altman plots indicate that the predicted joint flexions have a moderate LoA and a statistically significant difference from the gold-standard, Table 4 suggests the PCA can use discrete gait parameters reported by the markerless assessment system to generate feature models that are comparable to the ground truth, if there are sufficient samples.

## 4. Discussion and Conclusions

This paper introduced a markerless solution assisted by the BlazePose human pose detection model and a monocular camera for clinical gait analysis. In this study, we continue our previous research by implementing two optimisations for the gait signal processing stage and utilising a robust dataset, with more diverse samples provided by MoVi to validate the performance of markerless gait analysis system for processing video samples from indoor contexts with limited gait cycles.

According to the results of the experiment, the predicted gait signal accuracy is dependent on the performance of the human pose detection model. Although the post-processing part can filter some signal noises, the post-processing part will fail if the unexpected shape distortions occur in the original signal. In particular, we found that post-processing could not recover a reliable filtered ankle signal, as there was significant distortion in the original ankle signal shape. One possible reason for the original ankle signal distortion is that BlazePose’s estimation of foot location is more susceptible to obstruction than the knee or hip. To prevent incorrect ankle discrete gait parameters from disturbing the $T^{2}$ scores prediction, we excluded ankle-related gait signals from the system analysis.

**Figure 7:**
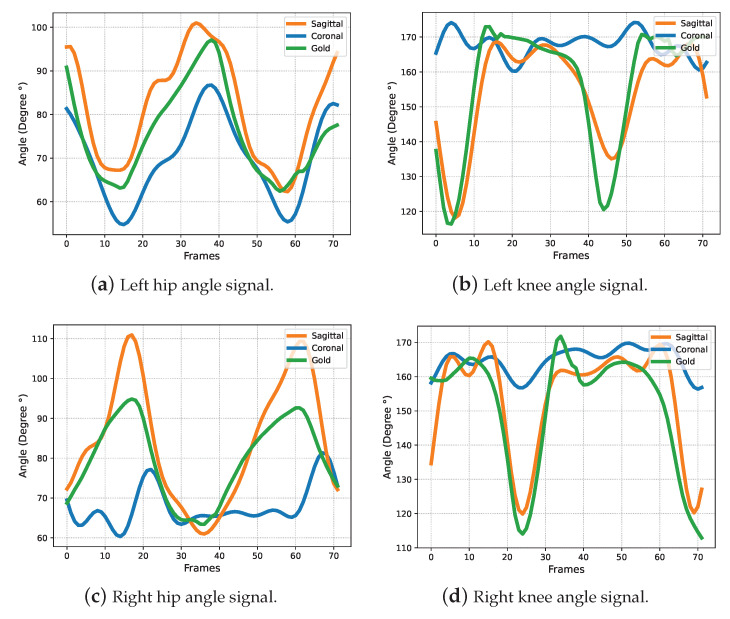
Comparison of gait signals (Subject 18) in the coronal plane, sagittal plane and ground truth.

To address the joint obstruction, we tried to feed BlazePose with a coronal plane rather than a sagittal plane video to compute the joint angle signal. However, comparisons in Figure 7 show that the joint angle signals obtained from the coronal plane tend to be distorted, which shows that it remains a challenge for the general human pose estimation model, BlazePose, to provide satisfactory 3D posture for medical rehabilitation application from a 2D coronal plane video. Therefore, sagittal plane videos are recommended inputs for our markerless gait analysis system. However, the innovation trends in computer vision and machine learning techniques are expected to provide better models for human pose estimation, which can cope with depth estimation in 2D video.

The experiment results also suggest the previous gait processing solution [15] has defects that caused the processed gait signal to suffer the worst PE and DTW distance. However, KF with two optimisations can significantly improve the 10.7% similarity of the predicted signals to the ground truth, without generating additional global offset compared with BlazePose’s raw prediction.

In addition, the Bland–Altman plots revealed that four joint flexion angles have 8.0% to 39.3% disagreement for prediction using the markerless method to the gold-standard using the traditional solution, while the 22.3% to 37.7% LoA will be an obstacle to calibrating the markerless assessment system’s results. However, the predicted female feature model shows a relatively good similarity to the gold-standard female feature model and the two methods’ $T^{2}$ scores have a r=0.99 and y=0.94x+0.01 regression line, which means that a markerless method can be a potential alternative to traditional solutions.

On the other hand, several limitations must be pointed out. For example, the current samples’ $T^{2}$ score clustered in the small interval makes it challenging for the Pearson correlation coefficient and the linear regression-based method to identify the deviation between the gold-standard and the predicted results. Another limitation is the absence of a comparison with related works, because the majority of validation work on gait analysis measurements employed independent, closed-source datasets [11,28,29], making it hard to obtain objective and fair comparisons from other related studies. This motivated us to utilise MoVi (public dataset) to validate the markerless system, which enables our work to provide a valuable assessment for subsequent proposed measurements in the gait analysis field.

To conclude, the markerless assessment system for indoor environments, which relies on BlazePose and a monocular camera, do not provide the same level of performance metrics compared to the marker-based methods, which is an obstacle to a fully independent clinical analysis task. However, from another perspective, the human pose detection model and a monocular RGB camera allow the assessment system to be freed from bulky and expensive professional data collection equipment and enable the system to complete preliminary clinical gait analysis on affordable personal devices, such as personal computers and smartphones. Moreover, the experiments support that the markerless method can be a potential alternative to traditional solutions, assisting with healthcare in clinical diagnosis by analysing the patient’s gait and returning the visualised reports, which supports us in developing an on-device application and analysing its computational consumption in future. To achieve a fully independent clinical analysis task, other strategies—for instance, recording two round walking videos with a different sagittal plane and combining them into a target’s gait signals—can be attempted in the future to achieve further improvements in accuracy.

## Figures and Tables

**Table 1:** Dataset’s basic information.

Subject	Gender	Age	Cut Interval	Subject	Gender	Age	Cut Interval
3	male	26	(900, 950)	48	female	18	(4700, 4760)
4	male	26	(1335, 1390)	49	female	23	(1700, 1810)
5	male	23	(836, 890)	50	female	18	(1900, 1990)
8	female	22	(2020, 2100)	51	female	18	(2360, 2420)
10	female	24	(680, 770)	53	female	23	(2550, 2650)
11	male	27	(4194, 4277)	54	female	18	(990, 1060)
12	female	26	(3465, 3535)	55	female	20	(4370, 4420)
13	male	26	(2365, 2420)	56	female	19	(3500, 3560)
15	male	21	(3460, 3530)	57	female	17	(640, 720)
16	female	26	(210, 280)	58	female	18	(3680, 3760)
17	female	26	(2590, 2660)	59	female	18	(3920, 3990)
18	male	25	(1132, 1212)	60	male	21	(2930, 3020)
19	male	18	(3250, 3320)	61	female	18	(1850, 1925)
20	male	29	(690, 760)	62	female	17	(3710, 3770)
22	male	28	(1218, 1284)	64	female	18	(3600, 3680)
23	male	25	(2095, 2140)	65	female	19	(3940, 4020)
24	female	20	(1130, 1220)	66	female	18	(2020, 2100)
25	female	21	(2920, 2970)	67	female	18	(4410, 4490)
26	male	24	(3690, 3780)	68	female	20	(2870, 2930)
27	male	23	(3465, 3552)	69	female	19	(1310, 1390)
28	male	25	(2605, 2675)	70	female	17	(820, 890)
30	female	19	(4310, 4380)	71	male	18	(360, 420)
31	male	28	(3305, 3375)	72	female	20	(3760, 3830)
32	female	20	(3740, 3805)	73	female	18	(500, 580)
33	male	21	(290, 350)	74	female	19	(2020, 2100)
34	female	21	(680, 740)	75	male	19	(1720, 1780)
35	male	29	(4508, 4588)	76	female	19	(3340, 3440)
36	male	29	(860, 920)	77	female	19	(1650, 1730)
37	male	21	(4610, 4690)	78	female	18	(730, 790)
38	female	32	(250, 350)	79	female	19	(3780, 3840)
39	female	21	(410, 475)	80	female	19	(2560, 2620)
40	female	21	(3866, 3950)	81	female	18	(3990, 4060)
41	male	28	(1860, 1910)	82	female	17	(2420, 2520)
42	male	21	(2020, 2080)	84	female	20	(3130, 3190)
43	male	21	(2460, 2540)	85	female	19	(2880, 2970)
44	female	20	(2710, 2770)	86	female	18	(2180, 2250)
45	female	18	(480, 550)	87	male	18	(1830, 1890)
46	male	21	(2960, 3040)	88	female	19	(3390, 3460)
47	male	18	(5200, 5255)	89	female	21	(3580, 3650)

**Table 2:** The performance of KF using different α and β combinations.

α	β	Far-Side (Knee & Hip)		Near-Side (Knee & Hip)	
		DTW	PE	DTW	PE
0.001	0.001	9.88	80.92%	5.39	45.37%
10	10	7.75	61.64%	5.30	44.89%
10	1	7.72	58.85%	5.36	42.86%
10	0.1	7.77	58.49%	5.38	42.57%
1	1	7.75	62.18%	5.31	44.99%
1	0.1	7.70	58.89%	5.36	42.88%
1	0.01	7.76	58.49%	5.38	42.57%
0.1	0.1	7.64	62.55%	5.34	45.18%
0.1	0.01	7.65	58.85%	5.37	42.91%
0.1	0.001	7.76	58.48%	5.38	42.57%
0.01	0.01	7.52	62.46%	5.37	45.32%
0.01	0.001	7.62	58.72%	5.37	42.94%
0.01	0.0001	7.75	58.46%	5.38	42.57%
0.001	0.001	7.50	62.16%	5.39	45.37%
0.001	0.0001	7.60	58.62%	5.38	42.95%

**Table 3:** Gait signals’ accuracy comparison for $KF_{1}$, $KF_{2}$, and Original signal.

Method	Far-Side				Near-Side				Average	
	Knee		Hip		Knee		Hip			
	DTW	PE	DTW	PE	DTW	PE	DTW	PE	DTW	PE
$KF_{1}+FDF$	5.08	36.11%	4.85	45.00%	2.91	18.27%	2.48	27.15%	3.83	31.63%
$KF_{2}+FDF$	4.17	25.81%	3.46	32.88%	2.94	17.33%	2.43	25.57%	3.25	25.40%
Original signal	4.59	25.93%	4.05	37.71%	2.95	17.90%	2.98	27.77%	3.64	27.32%

**Table 4:** PCs comparison for predicted feature model and gold-standard feature model.

Gender	PC	Explained Variance		Cosine Similarity
		Predictions	Gold-Standard	
Female	0	26.45%	30.11%	0.89
	1	18.64%	17.61%	0.78
	2	13.86%	16.07%	0.83
	3	12.13%	11.08%	0.71
				
Male	0	25.61%	38.12%	0.65
	1	21.55%	21.01%	0.72
	2	17.13%	13.42%	0.15
	3	12.20%	10.61%	0.13
